# The long-term effect of mTOR inhibition on lipid and glucose metabolism in tuberous sclerosis complex: data from the Dutch TSC registry

**DOI:** 10.1186/s13023-022-02385-8

**Published:** 2022-07-08

**Authors:** Femke V. M. Mulder, Evelien F. H. I. Peeters, Jan Westerink, Fried J. T. Zwartkruis, Wendela L. de Ranitz-Greven

**Affiliations:** 1 Department of Internal Medicine, University Medical Center Utrecht, Utrecht, The Netherlands; 2 dLAB and Center for Molecular Medicine, University Medical Center Utrecht, Utrecht, The Netherlands

**Keywords:** Tuberous sclerosis complex, mTOR inhibition, Dyslipidemia, Hypercholesterolemia, Hyperglycemia, Adverse effects, Long-term, Diabetes mellitus

## Abstract

**Background:**

MTOR inhibition is an effective treatment for many manifestations of tuberous sclerosis complex. Because mTOR inhibition is a disease modifying therapy, lifelong use will most likely be necessary. This study addresses the long-term effects of mTOR inhibitors on lipid and glucose metabolism and aims to provide better insight in the incidence and time course of these metabolic adverse effects in treated TSC patients.

**Methods:**

All patients who gave informed consent for the nationwide TSC Registry and were ever treated with mTOR inhibitors (sirolimus and/or everolimus) were included. Lipid profiles, HbA1c and medication were analysed in all patients before and during mTOR inhibitor treatment.

**Results:**

We included 141 patients, the median age was 36 years, median use of mTOR inhibitors 5.1 years (aimed serum levels 3.0–5.0 µg/l). Total cholesterol, LDL- and HDL-cholesterol levels at baseline were similar to healthy reference data. After start of mTOR inhibition therapy, total cholesterol, LDL-cholesterol and triglycerides increased significantly and were higher compared to healthy reference population. Mean total cholesterol levels increased by 1.0 mmol/L after 3–6 months of mTOR inhibition therapy but did not increase further during follow-up. In this study, 2.5% (3/118) of patients developed diabetes (defined as an HbA1c ≥ 48 mmol/mol) during a median follow-up of 5 years.

**Conclusions:**

Hypercholesterolemia is a frequent side effect of mTOR inhibition in TSC patients, and predominantly occurs within the first year of treatment. Although hyperglycemia is a frequent side effect in other indications for mTOR inhibition, incidence of diabetes mellitus in TSC patients was only 2.5%. This may reflect the difference of mTOR inhibition in patients with normal mTOR complex pathway function versus patients with overactive mTOR complex signaling due to a genetic defect (TSC patients).

## Background

Tuberous Sclerosis Complex (TSC) is a rare autosomal dominant genetic disorder, with reported prevalence rates from 0.7 to 10 per 100.000 [1]. The disease is caused by a mutation in either the TSC1 (hamartin) or TSC2 (tuberin) gene on chromosome 9 and 16, respectively. The mutations cause a dysregulation of the mechanistic Target of Rapamycin (mTOR) pathway that is involved in regulating cellular growth. Dysregulation of this pathway, has many different manifestations, including the development of tumours, such as renal angiomyolipomas (rAML) and subependymal giant-cell astrocytomas (SEGA).

MTOR inhibitors (mTORi) are very effective in treating rAML (> 30% size reduction after 4–5 years in more than 80% of patients), SEGA (response rate 100%) and epilepsy (response rate 40%) [2–6].

MTOR is part of a complex signaling network, playing a key role in several cellular processes regulating cell growth and metabolism. The TSC1/2 heterodimer normally has an inhibitory effect on mTOR complex 1 (mTORC1). Due to the malfunctioning of this dimer (due to a mutation), mTORC1 is hyperactive, resulting in cellular overgrowth. Inhibition of the hyperactive mTOR pathway is expected to normalize these cellular processes and therefore explain efficacy of mTORi therapy in TSC patients [7].

Before implementation in TSC patients, mTOR inhibitors were already in use as immunosuppressants after transplantation and antineoplastic treatment in various cancers. From these indications, mTOR inhibitors are known to induce dyslipidemia in 25–76% and hyperglycemia in 13–50% of patients [8–10].

In the EXIST trials (follow-up up to 5 years), the phase III trials that led to approval of mTOR inhibition in TSC patients, dyslipidemia was reported in 5–30%, but no new cases of diabetes were reported [3, 4, 6]. In a small prospective study among mainly children with TSC using mTOR inhibition, dyslipidemia and hyperglycemia were reported in 72% and 22% of children, respectively, but the highest fasting glucose did not reach diabetic levels (108 mg/dl (6 mmol/l)) [11].

MTOR inhibition is the first available therapy targeting the underlying pathophysiology of TSC. MTOR inhibition is thought to be a disease modifier, as interrupting treatment after less than a year resulted in regrowth of the tumours [12]. Since lifelong use is the standard of care at the moment, it is important to further investigate the occurrence of side effects in the long term. Expanding knowledge about the baseline lipid profiles of TSC patients and the effect of mTOR inhibitors on lipid and glucose levels will benefit appropriate follow-up and treatment in order to improve long-term health outcomes.

## Patients and methods

### Study design and patient selection

The Dutch TSC registry, which started in 2019, enrolled all patients from University Medical Center Utrecht (UMCU) with a definite diagnosis of TSC according to consensus criteria [13, 14]. All patients in the TSC registry gave informed consent (n = 316). The Dutch TSC registry is a registry of data from electronical medical records. Data has been collected through conventional follow-up according to good clinical practice and international guidelines. Baseline characteristics, laboratory outcomes and medication data have been collected from the TSC Registry. Additional data was added from electronical medical records by the investigators. Laboratory outcomes were not routinely acquired in fasting conditions.

All patients in the TSC registry with an mTORi prescription before the 1^st^ of October, 2019 (n = 141), were included in this study. In case patients used mTOR inhibition for multiple periods (with an interruption of treatment of more than 3 months), only the first period of treatment has been included in this analysis. We included available data until 1st of February 2020.

MTORi dosages usually started at 5 or 10 mg daily (for everolimus), but follow-up dosages were variable since dosages were titrated to blood serum levels. For the majority of patients, serum levels were titrated between 3.0 and 5.0 µg/l. In only one patient, serum levels were titrated to a sustained higher level (10.0–15.0 µg/l, indication refractory epilepsy).

### Definitions

The effect of mTOR inhibitors on lipid metabolism was investigated by reviewing total cholesterol serum levels and use of lipid lowering medication (ATC code C10). Hypercholesterolemia was defined as a total cholesterol above 6.5 mmol/L, the upper limit of normal range (ULN) according to UMCU laboratory standards and corresponding to grade 1 adverse event. Hypercholesterolemia was graded by severity, according to Common terminology criteria for adverse events (CTCAE) [15].

The effect of mTOR inhibitors on glucose metabolism was investigated by reviewing Hemoglobin A1c (HbA1c) laboratory outcomes and prescription of blood glucose lowering agents (ATC code A10) in the study population without pre-existent diabetes. In this study population, (fasting) plasma glucose levels were not widely available and oral glucose tolerance testing (OGTT) was not performed. HbA1c levels were available in the majority of patients (n = 118), therefore, we defined an HbA1c ≥ 48 mmol/mol (≥ 6.5%) or use of glucose lowering medication as a diagnosis of diabetes mellitus[16]. This is in accordance with the criteria from the American Diabetes Association guidelines. (Patients can be diagnosed with diabetes mellitus, by either an HbA1c ≥ 48 mmol/mol (≥ 6.5%), fasting plasma glucose > 7.0 mmol/L or through OGTT [16].)

### Data analysis

For analysis of mean total cholesterol during follow-up, five time windows were defined: 3 years before until start of mTORi (baseline), 3–6 months after start, 1, 2 and 5 years (with a range of 0.5 year) after start of mTORi. If a time window included multiple values from one patient, the value closest to time point of interest (start, 6 months, 1, 2 and 5 years) was selected. Total cholesterol values in each time window were compared with baseline values, using paired samples t-test.

For analysis of baseline lipid profiles, all patients without use of lipid lowering medication and with full lipid profiles available before start of mTORi (up to 3 years before start) were selected (n = 51). The lipid profiles were compared to reference data from a Dutch healthy population, adjusted for age and sex [17, 18]. Non–high-density lipoprotein cholesterol (non–HDL-c) reference data were not available and therefore could not be included in this analysis. To compare baseline data to reference data (50^th^ percentile), one sample Wilcoxon signed-rank test was used. To compare with lipid profiles during treatment, the same patients were selected for the control group if full lipid profiles were available at baseline and during mTORi treatment (at any time between 3 months to 3 years after start of mTORi, n = 33). To compare data (absolute serum levels and percentiles) during treatment with baseline, related-samples Wilcoxon signed-rank test was used. In all analyses, measurements after start of lipid lowering medication were excluded. Data were analyzed using SPSS version 25.0.0.2 (IBM Corp., Armonk, NY).

## Results

### Patient characteristics

We included 141 patients with TSC who used mTOR inhibitors. The majority (124/141 patients) started their treatment with everolimus. 17/141 started with sirolimus, of which 15 switched to everolimus and 2 discontinued treatment. Median follow-up time was 5.1 years. A total of 120/141 (85%) patients still used everolimus on cut-off date. The median age at start of mTOR inhibition was 35 years (Table 1). Only one patient discontinued treatment because of metabolic side effects.

**Table 1:** Patient baseline characteristics (n = 141)

Characteristics	Median [range] or n (%)
Age at start mTORi (years)	35.8 [17–76]
Male sex	69 (48.9)
Mutation type	
TSC1	17 (12.1)
TSC2	63 (44.7)
VUS	1 (0.7)
No mutation identified	10 (7.1)
Unknown	50 (35.5)
rAML	127 (90.1)
SEGA	46 (32.6)
Epilepsy	120 (85)
LAM	37 (26.2)
Duration use mTORi (years)	5.1 [0.1–13.8]
mTORi type^a^	
Sirolimus	17
Everolimus	139

^a^15 patients used sirolimus before switching to everolimus. mTORi, mechanistic target of rapamycin inhibitor; VUS, variant of unknown significance; rAML, renal angiomyolipoma; LAM, lymphangioleiomyomatosis; SEGA, subependymal giant-cell astrocytoma

### Lipids

**Fig. 1:**
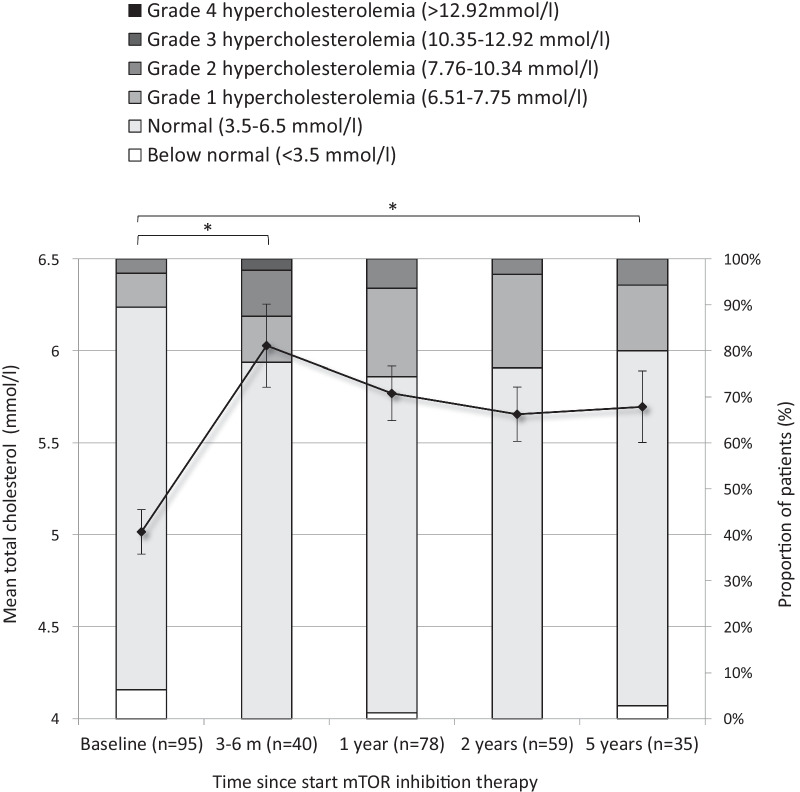
Mean and categorized total cholesterol before and during mTOR inhibition therapy. Error bar represents standard error of the mean. Total cholesterol values are clustered in categories according to CTCAE grading for adverse events. N represents number of patients from baseline population (n = 95) with available total cholesterol data in that time window. *p < 0.01

Mean total cholesterol levels increased from 5.0 mmol/L at baseline to 6.0 mmol/L at 3–6 months and 5.8 mmol/L at 1 year after start of mTORi (*p* < 0.01; Fig. 1). After 5 years of mTORi therapy, the mean total cholesterol remained stable. Before start of mTORi, 11% of the population had a total cholesterol > 6.5 mmol/L (upper limit of normal), which increased to 20–25% of the study cohort during treatment. CTCAE grade 3 total cholesterol levels were rare, and occurred only in 1 patient, none of the patients reached grade 4.

The proportion of patients using lipid lowering medication increased during mTORi use, progressing from 5% before, to 15.6% after 5-year follow-up. None of the patients stopped lipid lowering therapy at any point during mTORi treatment.

**Fig. 2:**
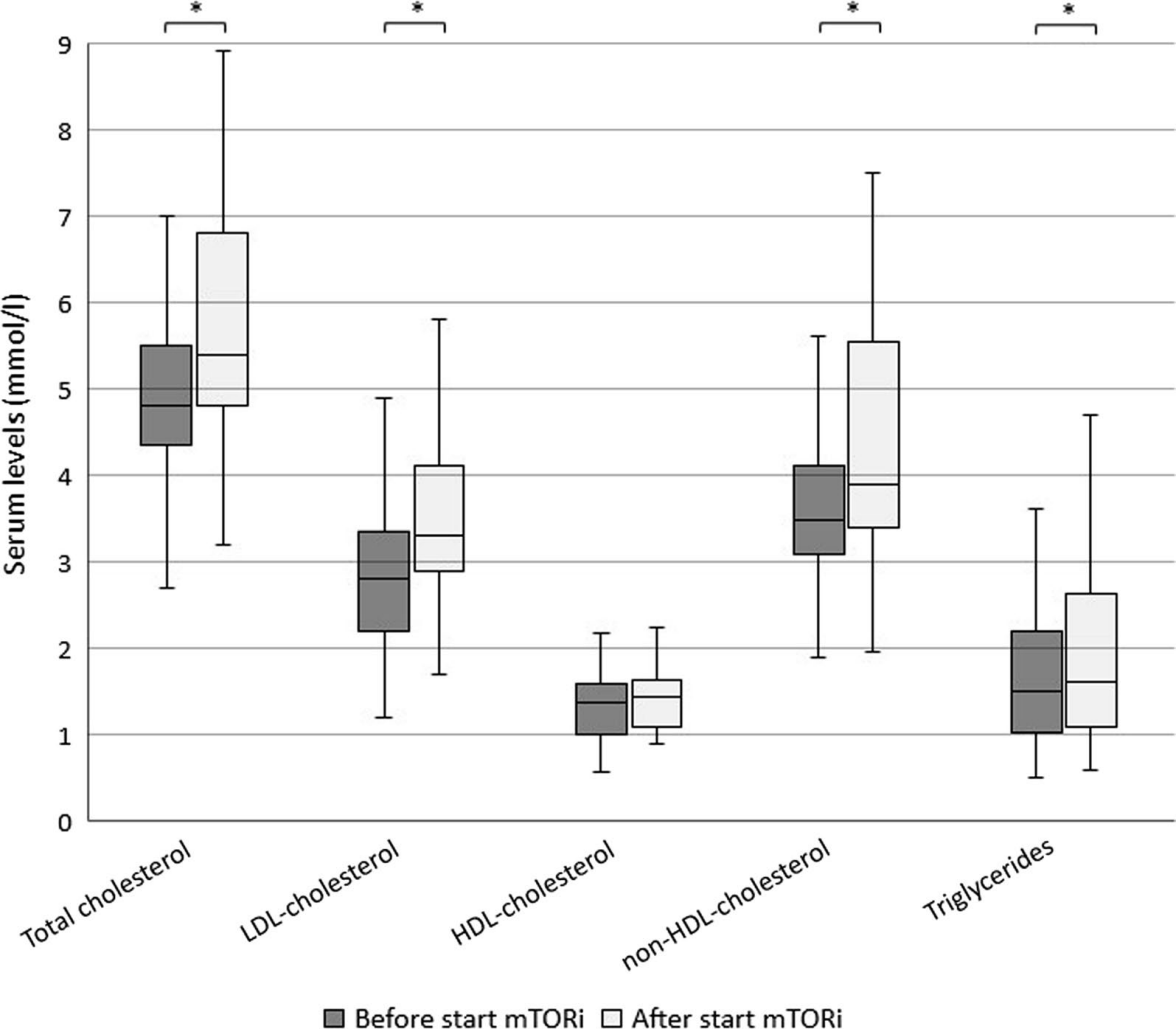
Boxplot of absolute serum levels of lipid profiles before and during mTOR inhibition therapy. Before start mTORi (3–0 years) N = 51, after start mTORi (3 months to 3 years) N = 33. *p < 0.01

Median total cholesterol, low density lipoprotein cholesterol (LDL-c), non-high density lipoprotein cholesterol (non-HDL-c) and triglycerides increased significantly after starting mTORi (*p* < 0.01; Fig. 2), while high density lipoprotein cholesterol (HDL-c) did not (*p* = 0.147).

**Fig. 3:**
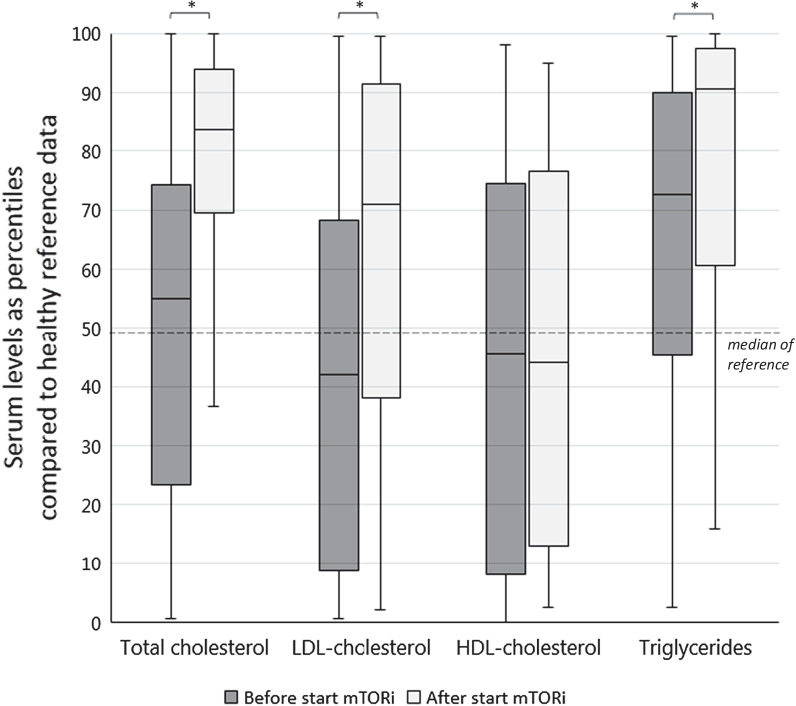
Boxplot of lipid profile before and during mTOR inhibition therapy, compared to healthy reference data. Before start mTORi (3–0 years) N = 51, after start mTORi (3 months to 3 years) N = 33. Data was expressed as percentiles compared to healthy (non TSC) reference population, adjusted for sex and age. The median of the healthy reference population equals the 50th percentile (thick line) *p < 0.05. Outliers not shown

Baseline lipid profiles in TSC patients were similar to healthy reference population (reference is age-matched 50th percentile) for total cholesterol (median percentile 55, *p* = 0.583); LDL-c (median percentile 42, *p* = 0.060) and HDL-c levels (median percentile 45.5, *p* = 0.117). An exception were the triglyceride levels, which were higher at baseline (median percentile 72.5, *p* < 0.01). After starting mTORi treatment, total cholesterol levels increased significantly (median percentile 55–83.5, *p* < 0.01), as well as LDL-c levels (median percentile 42–71, *p* < 0.01) and triglyceride levels (median percentile 72.5–90.5, *p* = 0.023). In contrast to HDL-c before and after starting mTORi therapy, which did not change (median percentile 45.5–44, *p* = 0.210) (Fig. 3).

### HbA1c

At baseline, 5/141 patients had pre-existent diabetes mellitus (3 patients with type 1 diabetes and 2 patients with type 2 diabetes). These patients were excluded for the analysis of incidence of diabetes during mTORi. After a median follow-up of 5.2 years [range 0.3–13.1], 2.5% of the patients developed diabetes during mTORi treatment, after 1.1, 1.2 and 8.9 years (HbA1c levels were 48, 49 and 56 mmol/mol). In addition, one patient developed HbA1c levels in the pre-diabetic range (HbA1c 42–48 mmol/mol). Reviewing medication lists of all 136 non-diabetic patients for newly prescribed glucose-lowering medication did not reveal any other new onset diabetes.

## Discussion

In the present study, we show that the use of mTOR inhibition in patients with TSC is associated with a significant and stable increase in the prevalence of hyperlipidemia. At the same time, the incidence of new cases of diabetes mellitus was 2.5%.

To compare our data to previous research, it is only possible to compare incidence of hypercholesterolemia (total cholesterol above upper limit of normal, > 6.5 mmol/L). The incidence of hypercholesterolemia in our study (20–25%), is consistent with data from the EXIST trials (5–30% after four years [3, 4]). The incidence is lower than the 72% reported by Trelinska et al. [11], although their small sample size limits comparability.

Our study clearly shows that dyslipidemia predominantly occurred in the first 3–6 months of treatment. Furthermore, our data show that the increase in cholesterol is permanent during median follow-up of 5 years. Whether lifestyle interventions, or dosage alterations impacted the data is unsure. Moreover, it must be noted that during follow-up, some patients dropped out of the analysis because of start of lipid lowering medication. Therefore, our research might even underestimate the actual effect of mTOR inhibition on cholesterol levels.

Our study is the first to compare baseline lipid profiles of TSC patients to healthy reference data, showing no significant differences, apart from triglycerides. This could be explained by the fact that lab testing was not routinely performed in fasting conditions, but an actual underlying difference in triglyceride metabolism in TSC patients is possible.

Analysing data as percentiles compared to healthy reference, benefits comparability while taking into account the effect of time (increasing age during follow-up). However, extreme lipid levels could be concealed in this method (a total cholesterol of 10 as well as 18 mmol/l would result in the 100^th^ percentile), but as shown in Fig. 1, extreme levels are rare in our population.

Our data shows that baseline lipid profiles do not differ significantly from healthy references. However, during treatment, total cholesterol, LDL-cholesterol and non-HDL cholesterol are significantly higher compared to reference data.

At first glance, the increase of cholesterol levels after mTORi initiation seems at odds with the well-known stimulatory role of mTOR on cholesterol synthesis via regulation of the transcription factor SREBP [19–21]. However, the combination of an increase in both total cholesterol, non-HDL-cholesterol, LDL-cholesterol and triglycerides points to an increase in apoB containing particles. This would be consistent with the described role of mTOR in inhibiting translation of apoB [22], although other mechanisms may be at play as well [23]. In the presence of insulin resistance this may be due to an increase in very low-density lipoprotein cholesterol (VLDL) and remnant production and reduced clearance. Further studies are necessary to investigate the precise changes in apolipoproteins and the atherogenicity of this profile.

Non-HDL-c has shown to be the most strongly associated with cardiovascular risk, with a trend in increased risk of 1.16 for an increase of 0.9 mmol/L [24]. In our population the median non-HDL-c increased from 3.5 to 3.9 mmol/L. Our results suggest that the effect is not temporary. Furthermore, mTORi therapy is expected to be used in younger age and treatment is usually long—term. Because lifelong mTORi therapy is frequently indicated, start of lipid lowering medication should be considered in the first year of treatment if hypercholesterolemia occurs. At the same time, if cholesterol is not elevated after 1 year of treatment, our data supports to stop regular cholesterol testing and only do so by indication.

In addition, our study shows that diabetes mellitus is not a common adverse event (incidence of diabetes de novo 2.5% in our cohort, with median follow-up time of more than five years). This percentage is much lower than one would expect based on 13–50% incidence of hyperglycemia in trials with mTORi as anti-cancer treatment in non-TSC patients [25].

However, the comparison to other studies is clearly limited due to differences in methods. All other studies that report hyperglycemia as an adverse event, did so by reviewing fasting glucose levels according to CTCAE guidelines [15]. These laboratory results were not available in our retrospective cohort, so no comparison can be made. However, the fact that elevated HbA1c levels (> 48 mmol/mol) did occur very infrequently in our population, does correspond with the absence of reported hyperglycemia in the EXIST-trials, and the modestly (non-diabetic) elevated glucose levels reported by Trelinska et al. [11].

Although not comparable to previous data, HbA1c levels in our study do provide relevant insight for clinical implications, as chronic hyperglycemia is clinically more relevant than a temporarily mild to moderate raised fasting blood glucose.

Interestingly, as hypothesised by Laplante & Sabatini and others [25, 26], hyperactivation as well as hypoactivation of mTOR is thought to be the cause for hyperglycemia. This hypothesis might explain why hyperglycemia does not occur in TSC patients, because the pathophysiological hyperactivation of mTORi in these patients is modulated by mTOR inhibition. This pathophysiological mechanism of mTORi treatment differs from the (theoretical) mechanisms in non-TSC patients. Another explanation could be the variance in dosages per indication. For example, the dosage of mTORi was10mg daily for the indication of renal cell carcinoma [9], whereas starting dosages for TSC patients in our study was usually 5 mg daily, subsequently titrated based on serum levels. A third hypothesis could be that in other indications (malignancies), mTOR inhibitors are combined with other antineoplastic agents that can increase the incidence of diabetes (for instance steroids). As far as data is available on these combination therapies, everolimus compared to placebo is independently associated with a higher incidence of hyperglycemia [25].

Strong aspects of our study are the large sample size and long-term follow-up. Since it is a retrospective study, some limitations are evident, such as missing a control group and missing values in laboratory results due to retrospective design. Furthermore, our data were not sufficient to perform analysis on the incidence of cardiovascular events in this population.

## Conclusions

In summary, hypercholesterolemia is a frequent adverse effect of mTOR inhibition in TSC patients, which occurs mainly in the first year of treatment. Therefore, as already stated in guidelines [27], evaluation of lipid profile in the first year of treatment is advised. Based on our study, regular follow-up of cholesterol after the first year of treatment, is unnecessary. As the incidence of diabetes mellitus in TSC patients on mTOR inhibition is only 2.5% in 5 years, we think this should open the debate if regular screening is warranted in this population. Further research should be performed to gain a better understanding of the pathophysiology of these adverse effects and associated risks in case of long-term use.

## Data Availability

The data that support the findings of this study are available from the Dutch TSC registry, but restrictions apply to the availability of these data, which were used under license for the current study, and so are not publicly available. Data are however available from the authors upon reasonable request and with permission of the Dutch TSC registry.
